# A Two-Stage Location-Allocation Optimization Method for Fixed UAV Nests in Power Inspection Considering Node Failure Scenarios

**DOI:** 10.3390/s25041089

**Published:** 2025-02-12

**Authors:** Zheng Huang, Hongxing Wang, Yiming Tang, Feng Gao, Biao Du, Jia Wang

**Affiliations:** 1 State Grid Jiangsu Electric Power Co., Ltd., Nanjing 210024, China; huangzhg10@163.com (Z.H.); whxseu@163.com (H.W.); ymtang@139.com (Y.T.); 2 School of Transportation Science and Engineering, Beihang University, Beijing 100191, China; jiawang@buaa.edu.cn; 3 Jiangsu Frontier Electric Technology Co., Ltd., Nanjing 211102, China; dubiao@js.sgcc.com.cn

**Keywords:** unmanned aerial vehicles, power facilities inspection, integer linear programming, UAV nest location problem, robustness

## Abstract

This paper explores the configuration and deployment of UAV nests for power inspection operations, focusing on potential nest failures. It proposes a two-stage location-allocation method. The problem is divided into two subproblems, each modeled as an integer linear programming (ILP) problem. The first subproblem identifies the minimal set of nodes for nest construction using the commercial solver Gurobi. The second subproblem involves UAV nest type selection and task allocation, solved with an ILS-SA heuristic algorithm. A case study in China shows that our method reduces total costs by 33.9% and decreases the number of UAV nests by 32% compared to the current greedy deployment method used by the power grid company. These results demonstrate the effectiveness and practicality of our approach in improving the reliability and cost-efficiency of UAV-based power inspection systems.

## 1. Introduction

The development of the power grid is fundamental to modern infrastructure, supporting the growth and progress of global societies. In recent decades, the grid has significantly expanded, driven by rising electricity demand and rapid industrialization and urbanization [1,2]. Regular power inspections are crucial for ensuring the proper functioning of the power grid. Traditional power inspections are conducted manually, resulting in low efficiency and safety hazards. UAVs, equipped with high-resolution cameras and sensors, can perform detailed inspections of power lines, identify potential faults, and enhance grid maintenance and safety. The integration of technologies such as artificial intelligence, cloud computing, big data, IoT, and 5G is driving the intelligent and autonomous development of UAV-based power inspections, laying a foundation for more efficient power grid management systems [3,4,5]. The application of UAV technology in power inspection systems, combined with advanced technologies like remote sensing, infrared, and data processing, has significantly enhanced the efficiency, accuracy, and safety of power line maintenance [6,7,8]. During power inspection tasks, UAVs utilize designated fixed UAV nests for takeoff, landing, charging, and maintenance. These nests, typically located near transmission towers, enhance operational efficiency and reduce inspection time. Therefore, the location of these UAV nests is critical, impacting the inspection process’s efficiency, coverage area, and emergency response times. The optimal deployment strategy of UAV nests can be regarded as a novel type of location optimization problem. Current research on the siting of UAV nests primarily models as set cover problems and p-median problems.

Set cover problems are a class of integer programming problems aiming to select the smallest number of subsets that together cover all elements of a larger set. Zhong et al. proposed a method that combines route planning and UAV airport site selection into a unified multi-objective optimization model, using redesigned particle swarm optimization strategies, effectively improving UAV airport utilization rates [9]. Park et al. modeled the problem of providing a wireless network in a disaster area using a UAV as a set cover problem, considering the operational constraints and advantages of UAVs. They proposed a branch-and-price algorithm to solve the coverage problem with two types of constraints [10]. Shakhatreh et al. addressed the problem of minimizing the number of UAVs needed for continuous coverage in disaster-stricken areas by developing the “cycles with limited energy” algorithm. This algorithm efficiently partitions the coverage area into recharge–station–starting cycles, significantly reducing the number of additional UAVs required compared to a straightforward method, especially as UAV energy capacity and the number of subareas increase [11]. Semiz and Polat tackled the multi-objective challenge of securing large areas with UAVs over a specific time window by developing a novel clustering-based algorithm, SVA, that solves a vehicle routing problem with time windows (VRPTW) variation [12]. Avellar et al. proposed a solution for achieving the shortest time ground coverage using a fleet of UAVs equipped with image sensors. Their method automatically selects the optimal number of UAVs based on flight and setup times [13]. Ayöperken and Ermiş transformed the problem of determining the optimal location for UAV bases into a facility location problem. They used a 0–1 binary integer programming (BIP) model, incorporating the idea of set coverage while considering target priorities, site constraints, and the heterogeneity of UAVs [14]. Di Puglia Pugliese et al. proposed mixed integer non-linear optimization models and heuristic procedures based on restricted mixed integer programming (MIP) to solve the mobile target coverage problem for UAVs with limited initial energy [15]. Zhang and Duan addressed the rapid deployment problem of UAVs by studying the minimization of the maximum deployment delay among all UAVs for fairness and the minimization of the total deployment delay for efficiency. They proposed an optimal algorithm with low computational complexity for the min-max problem [16]. Jing et al. proposed a multi-UAV coverage path planning framework that combines the set cover problem with the vehicle routing problem (VRP) to formulate a set cover vehicle routing problem [17]. Yuan et al. proposed an improved genetic algorithm to address the increased energy consumption issue in fixed-wing UAVs during area coverage tasks in specific scenarios, which arises due to inefficient path planning [18]. Shakhatreh et al. tackled the challenge of providing wireless coverage to indoor users in high-rise buildings during disasters with a single UAV. They proposed a realistic outdoor–indoor path loss model, studied the efficient UAV placement problem to minimize total transmit power, and offered solutions for two practical cases involving maximum path loss coverage and symmetric indoor user locations [19].

The p-median problem seeks to determine the optimal locations for p facilities to minimize the distance between clients and their nearest facility. Mozaffari et al. proposed a method for UAVs to move and change positions based on the distribution of users, solving the problem using the p-median facility location modeling framework [20]. Zahedi et al. modeled the placement problem of drone base stations (DBSs) as a p-median optimization problem. They used a fuzzy clustering algorithm to determine candidate locations for DBSs and solved the optimization problem to find the optimal number of antennas and the best positions for DBSs using the bisection method [21]. Sobouti et al. addressed the deployment problem of UAVs in the desired environment as a mathematical optimization model based on the p-median framework. They used dynamic channel allocation or dynamic frequency selection methods to avoid interference [22]. Shakhatreh et al. studied the deployment problem of a single UAV providing wireless coverage for users uniformly distributed within a high-rise building. Their goal was to minimize total transmission power while meeting indoor users’ rate requirements, using particle swarm optimization [23]. Savkin and Huang considered the problem of minimizing the average distance between UAVs and users while maintaining the connection between UAVs and fixed base stations to maximize overall service quality [24]. Wang et al. proposed an energy-saving deployment algorithm for UAVs that uses the minimum transmission power to serve a group of ground users while ensuring that the QoS requirements of edge users are met [25]. Noh et al. developed an energy-efficient deployment method for multiple UAVs using ellipse clustering to establish base stations. They adjusted the antenna’s half-power beamwidth, direction, and three-dimensional deployment position to minimize path loss for edge users, thus obtaining the coverage range of the UAV [26]. Bahr et al. applied the particle swarm optimization algorithm to address the problem of providing coverage for IoT devices using a wireless-enabled UAV in emergency communication scenarios. This approach focused on power-aware 3D UAV placement for IoT emergency communications [27]. Dai et al. introduced a multi-objective optimization approach utilizing GIS and TOPSIS for pre-screening, along with a greedy algorithm and Lagrange relaxation for the p-median coverage problem, to optimize UAV nest site selection and coordination, reducing inspection costs by over 9.2% [28].

Indeed, deploying UAV nests to achieve automated inspections can reduce labor costs and improve inspection safety and efficiency. However, the operation of UAV nests requires reliable power supply, stable network connections, and specific weather conditions, making it difficult to ensure long-term stability and reliability after deployment. Existing studies often assume ideal operating conditions, which can render deployment plans infeasible in the event of UAV nest failures. Additionally, most studies overlook the inspection frequency requirements of the power facility, thereby lacking a comprehensive analysis of the trade-offs between the construction costs of UAV nests and the daily inspection costs. Finally, existing research often focuses on the deployment of a single type of UAV nest and uses random methods to generate test cases, lacking applicability to large-scale real-world inspection scenarios. Considering the deficiencies in current research, the key contribution of this paper is summarized as follows:

- Considering the possibility of UAV nest failures, this paper proposes a two-stage configuration and deployment method for UAV nests in the inspection of transmission towers.
- The problem is decomposed into two subproblems, each modeled as an integer linear programming model.
- An iterated local search with a simulated annealing (ILS-SA) heuristic algorithm is proposed to solve the UAV nest type selection and task allocation scheme.
- Using a large-scale area in China as a case study, the proposed method was validated, demonstrating a 33.9% reduction in total costs and a 32% decrease in the number of UAV nests used compared to the greedy deployment method currently used by the power grid company.

The remaining of this paper is organized as follows: Section 2 describes the problem and formulates two ILP models for corresponding subproblems. Section 3 provides a detailed description of the proposed ILS-SA algorithm, while Section 4 presents the results of the computational experiment applied to a case of power inspection in China. A sensitivity analysis demonstrates the impact of changes in inspection field scenario parameters on UAV nest deployment scenarios. Section 5 concludes the paper.

## 2. Problem Description and Modeling

### 2.1. Problem Description

In a specific area, there are several transmission towers that require inspection. This paper investigates the deployment of UAV nests within the area to achieve automated UAV inspections of power facilities. Each UAV nest is paired with a UAV responsible for inspecting several transmission towers within the coverage area of the nest. During each inspection task, the UAV departs from the UAV nest after being fully charged, proceeds to the designated transmission towers, and returns to the UAV nest to recharge and await the next task upon completing the inspection.

**Figure 1:**
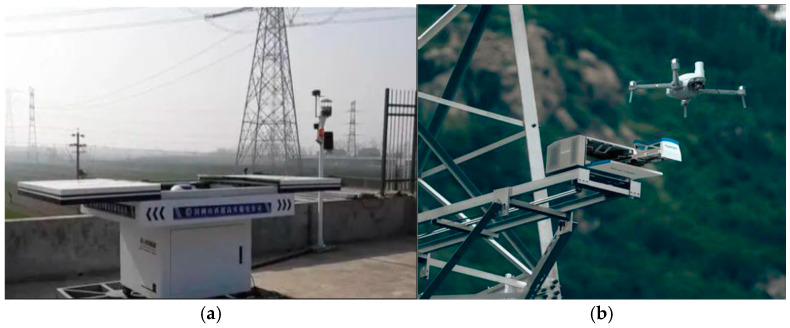
Two types of UAV nests: (**a**) reliable UAV nests and (**b**) unreliable UAV nests.

The selection of UAV nest locations is a strategic task. Once determined, the locations are not easily altered. Due to constraints such as power supply, signal, network, and security factors, UAV nests can only be deployed near transmission towers. In actual operation, UAV nests may fail due to weather conditions, mechanical failures, and other reasons, significantly impacting power line inspections. This paper assumes two types of UAV nests: reliable and unreliable, as shown in Figure 1. Reliable nests have a complex structure with high waterproof and dustproof ratings, typically placed in designated areas near transmission towers and regularly maintained by personnel. Therefore, we assume they remain operational throughout the inspection period. In contrast, unreliable nests are simpler, with lower protection levels, mounted directly on transmission towers, which results in lower costs but makes them more prone to failure and difficult to repair. As such, this paper assume a certain probability of failure, with the associated inspection tasks needing to be performed manually in case of failure. It is important to note that failure probabilities are used to calculate the expected performance of the system, rather than assuming that the entire cycle remains in a failed state after a failure occurs. This setup reasonably reflects the differences in construction and maintenance between the two types of nests. Due to their similar communication capabilities, the two types of nests are assigned the same coverage radius.

To address the large-scale automated UAV inspection problem encountered in practical applications, this paper designs a hierarchical solution process. By decomposing the complex overall problem into smaller, more manageable subproblems, the computational complexity at each level is effectively reduced, thereby improving solution efficiency. In the practical problem of UAV nest deployment for inspections, the construction cost of the nests constitutes a significant portion of the total expenses. Therefore, decision-makers often aim to use the minimum number of nests to achieve full coverage of all transmission towers within the inspection area. Subsequently, based on factors such as inspection frequency of the transmission towers [29], the distance between towers and nests, nest reliability, and manual inspection costs, they select the types of nests to use and determine the assignment of inspection tasks. Hence, this paper establishes a two-stage UAV nest location-allocation optimization model. The objective is to minimize the total cost by determining the deployment locations, types of nests, and the allocation of inspection demand points among the nests. This problem can be divided into the following two subproblems:

1. UAV nest construction site selection;
2. UAV nest type decision and task allocation scheme.

Subproblem 1 addresses the selection of candidate sites for UAV nests. By employing the minimum set cover problem, an integer linear programming (ILP) model is established with the objective of minimizing the number of UAV nests. The Gurobi solver is used to precisely solve the candidate site selection for UAV nests.

After determining several points from the candidate sites to be used as UAV nest construction nodes, subproblem 2 involves decision-making regarding the types of UAV nests and the task allocation scheme. The deployment of both reliable and unreliable UAV nests at the selected construction nodes is considered. The combined deployment of these two types of nests is intended to accomplish all inspection tasks within the area at the lowest total cost. The potential failure of unreliable nests is considered to make the model more realistic. An integer linear programming model is established, and a solution for this subproblem is designed using an iterated local search combined with simulated annealing (ILS-SA) algorithm. This approach aims to determine the optimal UAV nest types and task allocation scheme. Model parameters and variables are shown in Table 1 and Table 2.

Based on the practical inspection requirements, the following assumptions are made:

1. The maximum operational coverage range of reliable and unreliable UAV nests is the same. They can only provide inspection services if the task points to be inspected are within their coverage range;
2. The geographical locations of all transmission towers requiring inspection are known, and the distances between any two points are known;
3. Each UAV can complete the inspection task of only one tower per mission and must depart from and return to the corresponding UAV nest;
4. If an unreliable UAV nest fails, the UAV cannot depart from that nest;
5. The events of UAV nest failures are assumed to be independent;
6. The flight distance of the UAV is considered as the Euclidean distance in two dimensions, without accounting for flight altitude.

### 2.2. UAV Nest Construction Site Selection Model

The site selection for UAV nests is the first subproblem. Specifically, this is a set cover problem, with the objective of minimizing the number of UAV nests while ensuring that each inspection task point is covered by at least one nest. The objective function for the first subproblem is as follows:

(1) $$\min\sum_{j\in J}x_{j}$$

Subject to:

(2) $$\sum_{j\in J}X_{ij}\geq 1,\;\forall i\in I$$

(3) $$x_{j}\geq X_{ij},\;\forall i\in I,\;j\in J$$

In the model, Equation (1) represents the objective function, which aims to minimize the number of UAV nest construction sites, i.e., to establish the minimum number of nests required to accomplish the inspection tasks for all transmission towers within the area. Minimizing the number of UAV nests and the total cost is intended to balance the relationship between nest construction and operational costs with the actual inspection requirements.

The constraints are explained as follows: Equation (2) ensures that for any transmission tower i∈I requiring inspection, it must be within the coverage range of at least one UAV nest j∈J; Equation (3) ensures that a transmission tower i∈I can only be inspected by a UAV departing from nest j∈J if a nest is established at site j∈J.

### 2.3. UAV Nest Type Decision and Task Allocation Model

The UAV nest type decision and task allocation problem constitutes the second subproblem. Specifically, by comprehensively considering nest construction costs, inspection costs, and potential manual inspection costs in the event of nest failures, the objective is to minimize the total cost while ensuring that the inspection requirements of all task points are met. The objective function for the second subproblem is as follows:

(4) $$\min\sum_{j\in J*}\left(c_{1}y_{j}+c_{0}z_{j}\right)+T\sum_{i\in I}\sum_{j\in J*}f_{i}\alpha d_{ij}Y_{ij}+\sum_{i\in U}\sum_{j\in J*}f_{i}((1-P_{fail})\alpha d_{ij}Z_{ij}+P_{fail}\beta \varphi_{i}Z_{ij})$$

Subject to:

(5) $$\sum_{j\in J*}y_{j}\leq N_{1}$$

(6) $$\sum_{j\in J*}z_{j}\leq N_{0}$$

(7) $$\sum_{j\in J*}(Y_{ij}+Z_{ij})=1,\;\forall i\in I$$

(8) $$d_{ij}Y_{ij}\leq R,\;\forall i\in I,\;j\in J*$$

(9) $$d_{ij}Z_{ij}\leq R,\;\forall i\in I,\;j\in J*$$

(10) $$Y_{ij}\leq y_{j},\;\forall i\in I,\;j\in J*$$

(11) $$Z_{ij}\leq z_{j},\;\forall i\in I,\;j\in J*$$

In the model, Equation (4) represents the objective function aimed at minimizing the total cost, which consists of three parts. The first part represents the construction costs of the two types of UAV nests; the second part represents the total inspection cost for transmission towers assigned to reliable UAV nests within the specified inspection period; and the third part represents the total inspection cost for transmission towers assigned to unreliable UAV nests within the specified inspection period, including the total cost of UAV inspections under normal conditions and the total cost of manual inspections in case of nest failures.

The constraints are explained as follows:

Equation (5) limits the maximum number of reliable UAV nests that can be constructed. Equation (6) limits the maximum number of unreliable UAV nests that can be constructed. Equation (7) ensures that each transmission tower requiring inspection is served by exactly one reliable or unreliable UAV nest. Equations (8) and (9) represent the operational radius constraints for the two types of UAV nests. Equation (10) ensures that a construction site that does not have a reliable UAV nest cannot provide inspection services for the corresponding transmission tower. Equation (11) ensures that a construction site that does not have an unreliable UAV nest cannot provide inspection services for the corresponding transmission tower.

## 3. Algorithm Design

### 3.1. The Solution of UAV Nest Construction Site Selection

The Gurobi solver is employed to solve the first subproblem, providing the exact solution for the minimal set of nest construction points, denoted as set J*.

### 3.2. ILS-SA

This section introduces an iterated local search with simulated annealing (ILS-SA) metaheuristic algorithm to address the second subproblem. This subproblem combines UAV nest type decision-making with task allocation between the inspection tower and the nest. Therefore, a solution to this subproblem comprises these two components. The ILS-SA algorithm proposed in this paper is based on a set of neighborhoods, where each neighborhood represents a set of solutions obtainable from the current solution by applying some specified operations. By iteratively selecting neighborhoods and using the acceptance criteria of simulated annealing, the quality of the solution is continuously improved.

#### 3.2.1. The Neighborhood Operator

For addressing the structure of the solution to this problem, three different neighborhood structures have been implemented.

**Figure 2:**
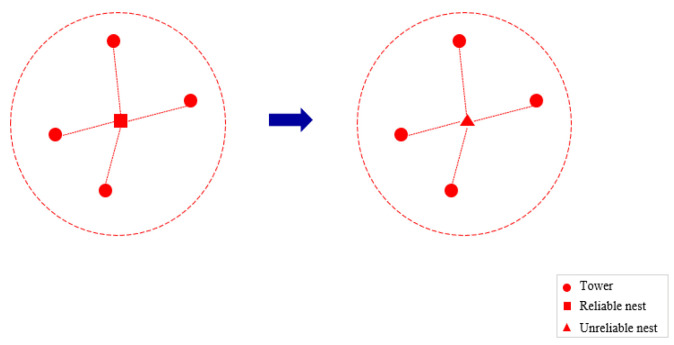
Nest-opt neighborhood.

Nest-opt neighborhood: This neighborhood involves randomly selecting a node from the nest construction points J*. If the current type of nest is a reliable nest, it is replaced with an unreliable nest, and vice versa, as shown in Figure 2. This neighborhood is used in the early stages of the algorithm to reduce nest construction costs by adjusting the nest types.

Tower-opt neighborhood: This neighborhood alters the matching between nests and transmission towers through the following three steps:

Step 1: select the transmission towers that fall within the coverage area of two or more nests, denoted as set $I^{\prime }$, with $\left|I^{\prime }\right|$ representing the number of elements in this set;

Step 2: randomly generate a natural number $Num\in [1,\left|I^{\prime }\right|]$;

Step 3: randomly choose Num nests from set $I^{\prime }$ and randomly reassign them to new nests that meet the coverage requirements.

**Figure 3:**
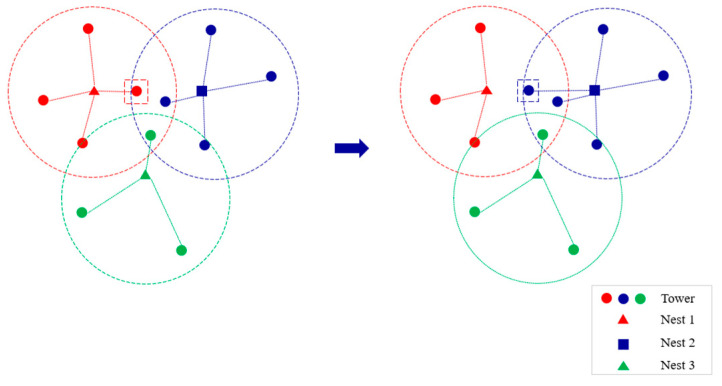
Tower-opt neighborhood.

The process is shown in Figure 3. This neighborhood is used in the mid-stage of iteration to reduce the UAV’s daily operational costs by adjusting the allocation of tasks between nests and towers.

Cross-exchange neighborhood: This neighborhood is a combination of the previous two neighborhoods. It involves performing the Nest-opt neighborhood and the Tower-opt neighborhood operations each once. This neighborhood is used in the mid and late stages of iteration to simultaneously adjust both nest types and the task allocation between nests and towers, thereby expanding the solution space and reducing the total cost.

#### 3.2.2. Initial Solution

A heuristic algorithm based on the greedy approach is designed to obtain the initial solution, which can be described as follows:

Step 1: Establish unreliable nests at all points in the nest construction set J* obtained from solving subproblem 1;

Step 2: Assign each tower in the set I to the nearest nest in the set J*.

#### 3.2.3. Simulated Annealing Acceptance Criterion

This paper employs the simulated annealing (SA) acceptance criterion, which is derived from the annealing process in metallurgy, simulating a gradual temperature reduction. During optimization, the ILS algorithm may become trapped in a local optimum, and the SA criterion allows the acceptance of a worse solution with a certain probability. This mechanism helps the algorithm escape from local optima, enabling broader exploration of the solution space and increasing the likelihood of finding a global optimum. The acceptance probability of SA decreases gradually with increasing iterations or decreasing temperature. At the early stages of the algorithm, the probability of accepting a worse solution is relatively high, which facilitates extensive exploration of the solution space and prevents premature convergence. In the later stages, the probability of accepting worse solutions decreases, allowing the algorithm to focus on exploiting the promising solutions found and gradually converging to a near-global optimum. This mechanism is widely applied in heuristic algorithms [30,31].

#### 3.2.4. ILS-SA Framework

This section illustrates the framework of the Algorithm 1: ILS-SA. The initial solution *s* is generated by a greedy algorithm (refer to Section 3.2.2 for details). Due to the significant proportion of nest construction costs in the total cost, the first neighborhood is used to generate new solutions in the early stages of iteration (when the number of iterations is less than 10% of the maximum iterations) (Lines 5 to 7). In the mid-stage of iteration (when the number of iterations is less than 50% of the maximum iterations), the second and third neighborhoods are used randomly to expand the search space while improving solution quality (Lines 8 to 14). In the later stages of iteration, the second neighborhood is used continuously to optimize the matching between nests and towers (Lines 15 to 17). As the algorithm tends to be trapped in a local minimum if it greedily accepts the solutions as long as they are better than the current best solution, simulated annealing (SA) acceptance criterion is applied here. Specifically, if *s^t^* is better than s*, then *s^t^* replaces both *s* and s* (Lines 18 to 20); otherwise, the SA criterion accepts the worse solution *s^t^* with particular probability and replaces *s* with *s^t^* (Lines 21 to 22). The entire ILS-SA algorithm terminates when the stopping criterion are met (Line 3), with an update to the iterative temperature $T_{SA}$ every iteration (Line 23).
**Algorithm 1:** ILS-SA
1: **Input** initial solution *s* found in Section 3.2.2; iterative temperature, $T_{SA}$; initial temperature, $T_{0}$; final temperature, $T_{f}$; cooling rate, γ; the number of global iterations, *Max_iter* 2: **Initialize**
s*←s; iter=0; $T=T_{0}$ 3: **While**
*iter < Max_iter*
**and**
$T\geq T_{f}$
**do** 4:    $s^{t}\leftarrow s$ 5:    **if** *iter < Max_iter* × 10% **then**
6:        $s^{t}\leftarrow NEIGHBOR_{1}(s)$ 7:    **end if** 8:    **elif** *iter < Max_iter* × 50% **then**
9:        **if** RANDOM (0,1) < 0.5 **then** 10:          $s^{t}\leftarrow NEIGHBOR_{2}(s)$ 11:        **else** 12:         $s^{t}\leftarrow NEIGHBOR_{3}(s)$ 13:        **end if** 14:    **end if** 15:    **else** 16:       $s^{t}\leftarrow NEIGHBOR_{2}(s)$ 17:    **end if** 18:    $\Delta =obj(s^{t})-obj(s*)$ 19:    **if**
Δ<0 **then**
20:       $s\leftarrow s^{t};\;\;\;s*\leftarrow s^{t}$ 21:    **elif** RANDOM (0,1) $\leq \;e^{-\Delta /T_{SA}}$ **then** 22:       $s\leftarrow s^{t}$ 23:    **end if** 24:    $T_{SA}\leftarrow T_{SA}\times \gamma$ 25:    *iter*++ 26: **end while** 27: **Return**
s*


## 4. Numerical Experiment

### 4.1. UAV Nest Construction Site Selection Case

**Figure 4:**
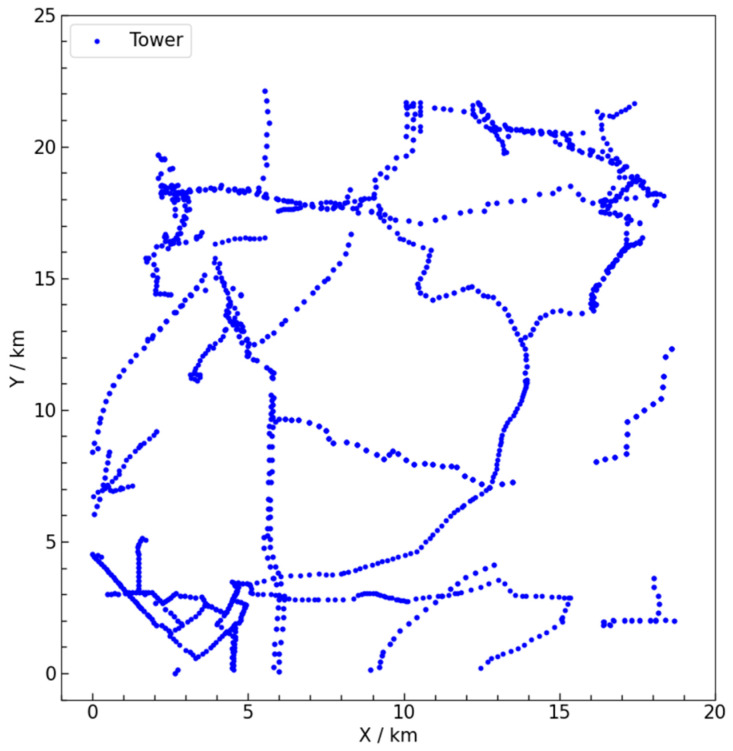
Study area and transmission tower distribution.

This paper utilizes actual data from transmission towers in a specific area of China. To reduce errors and protect data security and privacy, the original geographic coordinates (latitude and longitude) are converted to two-dimensional plane coordinates through geographic coordinate projection. By calculating the differences in longitude and latitude and using a transfer function to obtain the offsets in plane coordinates, the two-dimensional plane coordinates are derived using trigonometric functions. This projection ensures that the overall spatial distribution of the towers closely resembles the actual situation. An area of approximately 400 square kilometers is selected as the experimental case for algorithm validation and scenario comparison, as shown in Figure 4. In this figure, the X-axis and Y-axis units are in kilometers, and the coordinates represent the locations of the transmission towers. The transmission towers requiring inspection are marked with blue dots in the figure, totaling 2062 towers. The specific coordinate information for these towers is detailed in Table 3.

**Figure 5:**
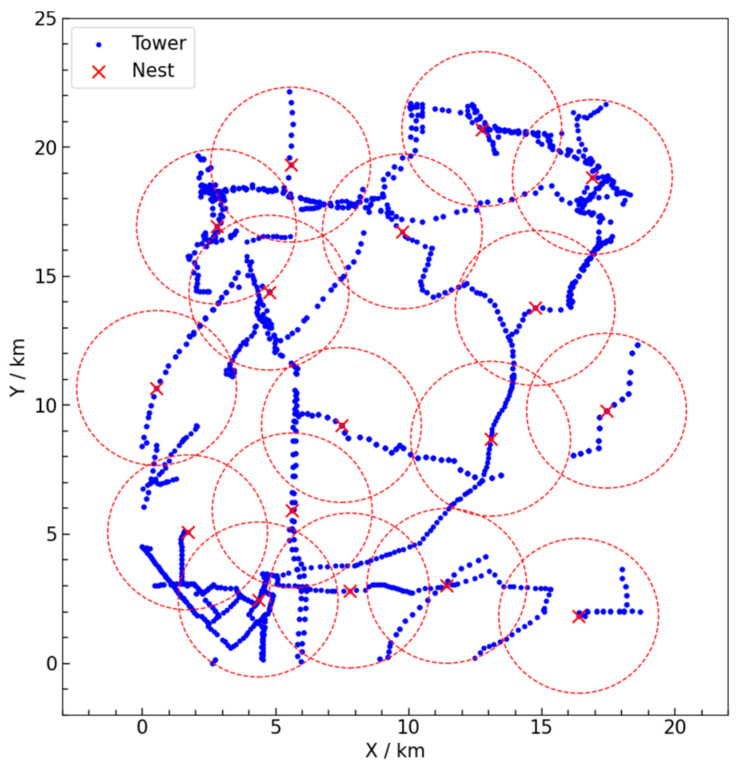
Candidate locations for UAV nests.

This study considers establishing several UAV nests within the aforementioned area to service the 2062 transmission towers requiring inspection. Each UAV nest can service all towers within a 3 km radius. The objective is to select the minimum number of transmission towers at which to establish UAV nests, ensuring that all towers needing inspection are covered by at least one UAV nest, thereby achieving the lowest construction cost. The Gurobi optimizer is employed to solve this problem. The solution obtained from the Gurobi optimizer identifies 17 optimal nest locations, as shown in Figure 5. The transmission towers requiring inspection are represented by blue dots, while the nest locations are indicated by red crosses, totaling 17 candidate points. The red dashed circles represent the coverage areas of the UAV nests. The specific coordinate information for the manual inspection starting point (Nest ID = 0) and the UAV nest candidate points (Nest ID = 1–17) is detailed in Table 4.

### 4.2. The Case of UAV Nest Type Selection and Task Allocation

**Figure 6:**
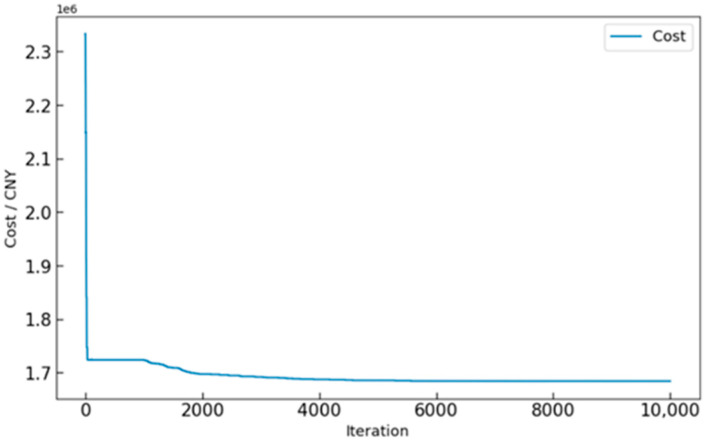
Cost convergence curve over iterations.

After obtaining the results for the UAV nest candidate points, ILS-SA is used to solve the robust UAV task allocation problem, resulting in the optimal UAV nest types and task allocation scheme. The algorithm parameters are detailed in Table 5. Throughout the algorithm’s execution, the cost value for each iteration was recorded, as illustrated in Figure 6. The horizontal axis represents the number of iterations, while the vertical axis represents the cost. It is evident that as the number of iterations increases, the cost value gradually decreases and stabilizes, indicating that the algorithm is progressively approaching the optimal solution.

**Figure 7:**
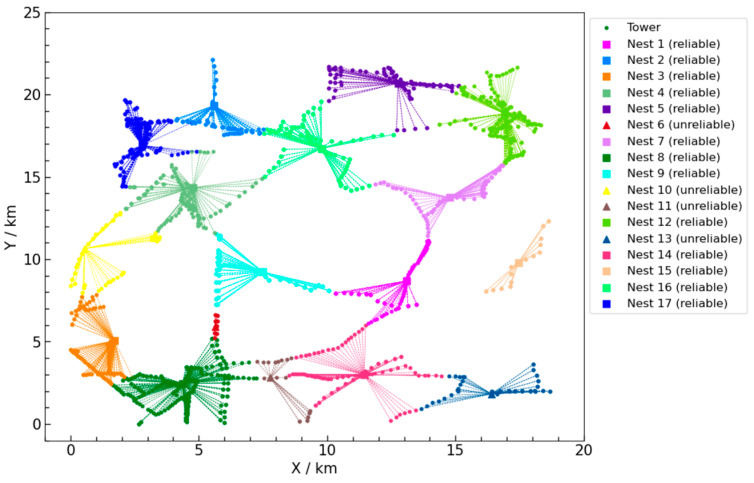
UAV nest type and task assignment scheme by ILS-SA algorithm.

The ILS-SA algorithm produced an optimal set of UAV nest deployments and their corresponding task allocation scheme, as depicted in Figure 7. Different colors of dots and lines represent different nests and their covered task points. Reliable nests and their corresponding task points are marked with squares, while unreliable nests are marked with triangles. Figure 4 includes a total of 13 reliable nests and 4 unreliable nests. The total cost of this scheme is 1,684,373 CNY. 

In the total cost of 1,684,373 CNY, the construction cost of UAV nests accounts for 91.5% (1,540,000 CNY), the UAV inspection cost accounts for 3.0% (51,223 CNY), and the manual inspection cost due to unreliable nest failures accounts for 5.5% (93,150 CNY). This indicates that construction cost is the primary cost component, emphasizing the need to optimize the number and configuration of UAV nests. Strategic site selection, reducing the number of reliable UAV nests, and appropriately deploying lower-cost unreliable nests are key optimization approaches. As the failure probability of unreliable UAV nests increases, the associated manual inspection cost also rises. Therefore, in regions with high inspection frequency and elevated failure risks, prioritizing the deployment of reliable UAV nests is essential to minimize long-term operational costs. In conclusion, deploying reliable UAV nests in critical areas while strategically utilizing unreliable nests in low-risk regions can effectively balance construction and operational costs, ultimately achieving the lowest total cost. An analysis of Figure 7 reveals that the UAV nest locations are relatively evenly distributed, and the task allocation scheme effectively covers all task points.

**Figure 8:**
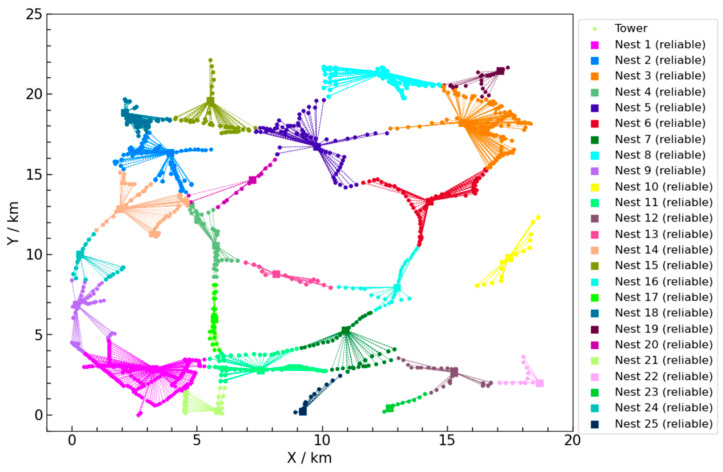
UAV nest type and task assignment scheme by greedy algorithm.

The performance of the improved algorithm was evaluated by comparing it with the classical greedy algorithm, which is commonly used for solving UAV nest location and task allocation problems. Existing research typically assumes that UAV nests do not experience failures, with the default assumption that nests are always in the normal operating condition. As a result, greedy algorithms tend to select solutions that exclusively involve reliable UAV nests. The UAV deployment and task allocation scheme obtained using the greedy algorithm is shown in Figure 8. The performance comparison between the ILS-SA algorithm and the greedy algorithm in solving the UAV nest location and task allocation problem is shown in Table 6. The main comparison metrics include total cost, the number of nests used, and the proportion of reliable nests. An analysis of Table 6 leads to the following conclusions:

1. Compared to the greedy algorithm, the ILS-SA algorithm reduces the total cost by approximately 33.9%. This indicates that the ILS-SA algorithm has a significant economic advantage and can utilize resources more efficiently;
2. The ILS-SA algorithm reduces the number of nests used by 8, a 32% reduction compared to the greedy algorithm. This not only lowers construction costs but also simplifies management and maintenance complexity;
3. In the ILS-SA algorithm, the proportion of reliable nests is 76%, whereas, in the greedy algorithm, the proportion is 100%. Currently, UAV inspection technology is still in the development stage, and a configuration entirely composed of reliable nests is not yet practical. There are still scenarios where unreliable nests and manual inspections are necessary.

By employing a mixed configuration of reliable and unreliable UAV nests, the ILS-SA algorithm not only reduces construction costs but also offers a flexible and scalable solution for UAV deployment. Reliable nests are costly but offer robust operational stability, making them suitable for critical inspection areas. However, due to their lower cost and easier installation, unreliable nests can effectively be used in less critical areas or for supplemental inspections, which reduces the overall economic burden. In practical operations, maintaining an entirely reliable configuration is often infeasible due to budgetary and logistical constraints, especially when considering the developmental stage of UAV inspection technology. This hybrid configuration helps in achieving an optimal balance between cost efficiency and inspection reliability, making it a more practical and adaptable solution compared to the fully reliable configuration suggested by the greedy algorithm.

Furthermore, unreliable nests, despite their higher failure rate, provide an opportunity to utilize manual inspections where needed, thereby maintaining coverage without excessive costs. This dual approach of utilizing both reliable and unreliable nests makes the ILS-SA solution more suitable for real-world conditions, where unpredictable variables like budget constraints and varying site criticality must be taken into account.

### 4.3. Sensitivity Analysis

A sensitivity analysis was conducted to further evaluate the robustness and effectiveness of the ILS-SA algorithm. This analysis mainly examines the algorithm’s performance under different parameter settings. The results of the sensitivity analysis provide a basis for optimizing parameter settings and demonstrate the algorithm’s adaptability in various scenarios.

#### 4.3.1. Sensitivity Analysis of the Probability of Failure to Unreliable UAV Nests ($P_{fail}$)

Multiple experiments were conducted to compare the total cost under different $P_{fail}$ settings, as presented in Table 7. It can be seen that as the failure probability $P_{fail}$ of unreliable UAV nests increases, the total cost shows an upward trend, and the proportion of reliable nests in the UAV nests also increases. This is mainly because a higher failure probability leads to more manual inspection costs.

#### 4.3.2. Sensitivity Analysis of the Total Operational Time *T*

The total cost was compared under different total operational times (*T*), as shown in Table 8. It can be seen that an increase in the inspection period *T* also leads to a rise in total cost, and the proportion of reliable nests in the UAV nests correspondingly increases. This indicates that, from a long-term perspective, UAV inspections are more cost-effective compared to manual inspections. Especially in scenarios with high nest failure probability and long inspection periods, choosing reliable nests can significantly reduce total costs.

#### 4.3.3. Sensitivity Analysis of the Cost of UAV Flight per Kilometer (α)

The total cost was compared under different cost of UAV flight per kilometer (α), as shown in Table 9. It can be observed that as α increases, the total cost exhibits an upward trend, while the UAV nest deployment scheme (i.e., the number of reliable and unreliable nests) remains unchanged. This indicates that variations in UAV inspection costs primarily affect operational expenses without influencing nest deployment decisions. The underlying reason is that UAV inspection costs constitute a relatively small proportion of the total cost (approximately 3%), making their impact on the final deployment strategy relatively limited.

#### 4.3.4. Sensitivity Analysis of the Cost of Manual Inspection per Kilometer (β)

The total cost was compared under different cost of manual inspection per kilometer (β), as shown in Table 10. It can be observed that as *β* increases, the total cost rises, accompanied by an increase in the number of reliable nests and a decrease in the number of unreliable nests. This indicates that higher manual inspection costs incentive decision-makers to deploy more reliable nests to mitigate manual inspection expenses. When *β* is relatively low (e.g., *β* = 0.25), the system tends to favor the deployment of lower-cost unreliable nests while accepting higher manual inspection costs. Conversely, when *β* is high (e.g., *β* = 2.00), the system completely eliminates the deployment of unreliable nests, making an all-reliable nest configuration more economically viable.

Compared to *α*, *β* has a more significant impact on the UAV nest deployment scheme, suggesting that manual inspection costs are the critical factor influencing UAV nest type selection. In contrast, UAV inspection costs (*α*) primarily affect long-term operational expenses, exerting minimal influence on nest siting and type selection.

## 5. Conclusions

This paper explores the location strategy for fixed UAV nests in power inspection operations. Considering potential nest failures, a two-stage location-allocation optimization method is designed. The problem is decomposed into two subproblems, each formulated as an ILP model. In the first stage, the commercial solver Gurobi is employed to obtain the exact solution for the minimal set of nest construction points. In the second stage, an ILS-SA heuristic algorithm is developed to determine the types of nests and their allocation schemes.

A case study is conducted using an operational maintenance area in China to validate the proposed method. The method’s performance is compared with that of a traditional greedy algorithm. Through the proposed two-stage UAV nest deployment and task allocation optimization method, the total cost was reduced by 33.9% compared to the greedy deployment method currently used by the power company, and the number of UAV nests was reduced by 32%. The results show that the improved method demonstrates higher effectiveness and practicality in solving the UAV nest location and task allocation problems.

In this study, we primarily considered the failure of UAV nests. However, future work could expand to more complex scenarios, such as considering the impact of uncertain factors like weather conditions and communication network instability on UAV inspections, and explore novel optimization methods to address these uncertainties. Furthermore, the algorithm presented in this paper has a certain level of generalizability. In future research, we plan to further explore and compare the applicability of the proposed algorithm in other fields (e.g., cellular relay station coverage, meteorological station placement, seismic station sensor deployment) to further verify its effectiveness and applicability in different application scenarios.

## Figures and Tables

**Table 1:** Definition of symbols.

Parameter	Definition
*I*	The set of power facilities (transmission towers) to be inspected, $I=\left\{1,2,\ldots ,\left|I\right|\right\}$, indexed by *i*.
*J*	The set of candidate UAV nest locations, This paper assumes that each transmission tower requiring inspection is a potential site for UAV nest deployment. Therefore, J=I, indexed by *j*.
J*	The set of construction points for the nests, i.e., the selected locations where UAV nests will be deployed.
*o*	Virtual nest: the starting point for manual inspections when the corresponding inspection tasks are taken over by human inspectors due to the failure of unreliable UAV nests.
$J^{\prime }$	The set of construction points and virtual nest, $J^{\prime }=J*\cup \{o\}$.
$d_{ij}$	The distance between the inspection transmission tower i∈I and the UAV nest, $J^{\prime }=J^{*}\cup \{o\}$, measured in km.
$f_{i}$	The inspection service demand frequency for the transmission tower i∈I to be inspected, measured in times per year.
*T*	The total operational time of the inspection system, measured in years.
$\varphi_{i}$	The penalty distance cost incurred when the transmission tower i∈I to be inspected by human inspectors, $\varphi_{i}=d_{io}$.
$N_{1}$	The maximum number of reliable UAV nests that can be constructed.
$N_{0}$	The maximum number of unreliable UAV nests that can be constructed.
$c_{1}$	The construction cost of a single reliable UAV nest.
$c_{0}$	The construction cost of a single unreliable UAV nest.
$P_{fail}$	The probability of failure to unreliable UAV nests.
α	The cost of UAV flight per kilometer.
β	The cost of manual inspection per kilometer when an unreliable UAV fails.
R	The maximum service radius of a UAV nest, measured in km.

**Table 2:** Decision variable.

Variables	Meaning
$x_{j}$	Boolean variable: takes the value 1 if a UAV nest is established at candidate site j∈J, otherwise 0.
$X_{ij}$	Boolean variable: takes the value 1 if the distance between candidate nest site j∈J and transmission tower i∈I is less than or equal to the UAV coverage range R, otherwise 0.
$y_{j}$	Boolean variable: takes the value 1 if a reliable UAV nest is established at the construction site j∈J*, otherwise 0.
$Y_{ij}$	Boolean variable: takes the value 1 if a reliable UAV nest at the construction site j∈J* provides inspection services for the transmission tower i∈I, otherwise 0.
$z_{j}$	Boolean variable: takes the value 1 if an unreliable UAV nest is established at the construction site j∈J*, otherwise 0.
$Z_{ij}$	Boolean variable: takes the value 1 if an unreliable UAV nest at the construction site j∈J* provides inspection services for the transmission tower i∈I, otherwise 0.

**Table 3:** Coordinate information of transmission tower.

Tower ID	X (km)	Y (km)
1	5.99	0.75
2	0.22	6.60
3	2.79	1.09
4	18.17	2.28
……		
2059	15.41	20.06
2060	11.69	7.84
2061	5.67	8.62
2062	4.30	2.32

**Table 4:** Coordinate information of the manual inspection starting point and the UAV nest candidate points.

Nest ID	Tower ID	X (km)	Y (km)	Nest ID	Tower ID	X (km)	Y (km)
0	0	−20	−20	9	1008	7.48	9.21
1	31	13.09	8.68	10	1208	0.54	10.64
2	78	5.58	19.30	11	1325	7.79	2.80
3	399	1.71	5.05	12	1352	16.91	18.82
4	565	4.76	14.34	13	1354	16.39	1.82
5	576	12.76	20.68	14	1584	11.44	2.98
6	663	5.64	5.90	15	1794	17.44	9.77
7	897	14.76	13.74	16	1814	9.77	16.72
8	910	4.36	2.45	17	1848	2.79	16.90

**Table 5:** Experimental parameters.

Parameters	Value
*R*/km	3
$c_{0}$/CNY	60,000
$c_{1}$/CNY	100,000
*P_fail_*	0.05
α/CNY	0.05
β/CNY	1
*f_i_*/times	52
*T*/years	3
*N*_0_	20
*N*_1_	20
*T*_0_	1000
*T_f_*	0.001
γ	0.99
*Max_iter*	10,000

**Table 6:** The comparison between ILS-SA and greedy algorithm.

Algorithm	Cost/CNY	Number of Nests	The Proportion of Reliable Nests
ILS-SA	1,684,373	17	76%
Greedy	2,548,282	25	100%

**Table 7:** The effect of the probability of failure to unreliable UAV nests $P_{fail}$ on the algorithm result (*T* = 3, α = 0.05, β = 1).

Variant	Value	Cost/CNY	Number of UAV Nests	
			Reliable Nest	Unreliable Nest
$P_{fail}$	0.02	1,519,002	5	12
	0.03	1,620,274	10	7
	0.05	1,684,373	13	4
	0.10	1,750,445	17	0

**Table 8:** The effect of the total operational time *T* on the algorithm result ($P_{fail}$ = 0.05, α = 0.05, β = 1).

Variant	Value	Cost/CNY	Number of UAV Nests	
			Reliable Nest	Unreliable Nest
*T*/years	1	1,430,367	3	14
	2	1,622,446	11	6
	3	1,684,373	13	4
	4	1,741,506	16	1

**Table 9:** The effect of the cost of UAV flight per kilometer α on the algorithm result ($P_{fail}$ = 0.05, *T* = 3, β = 1).

Variant	Value	Cost/CNY	Number of UAV Nests	
			Reliable Nest	Unreliable Nest
α	0.01	1,643,396	13	4
	0.05	1,684,373	13	4
	0.10	1,736,410	13	4
	0.20	1,838,965	13	4
	0.50	2,146,556	13	4

**Table 10:** The effect of the cost of manual inspection per kilometer β on the algorithm result ($P_{fail}$ = 0.05, *T* = 3, α = 0.05).

Variant	Value	Cost/CNY	Number of UAV Nests	
			Reliable Nest	Unreliable Nest
β	0.25	1,405,905	1	16
	0.5	1,562,593	7	10
	1	1,684,373	13	4
	1.5	1,726,319	16	1
	2	1,750,445	17	0

## Data Availability

Data are unavailable due to privacy restrictions.
